# BIOLOGICAL RISK PROFILES IN THE OLDER MEXICAN POPULATION

**DOI:** 10.1093/geroni/igz038.2900

**Published:** 2019-11-08

**Authors:** Catherine Garcia, Joseph Saenz, Jennifer A Ailshire, Rebecca Wong, Eileen M Crimmins

**Affiliations:** 1 University of Southern California, Los Angeles, California, United States; 2 University of Texas-Medical Branch, Galveston, Texas, United States

## Abstract

Research examining biological risk is critical given that both the Mexican and U.S. populations are aging. Biomarkers can help us understand underlying disease patterns among Mexican-origin individuals in Mexico and the U.S. to help inform disease-prevention efforts for these populations. Using data from the 2012 Mexican Health and Aging Study and the 2010/2012 Health and Retirement Study, we examine seven biomarkers known to predict health risk: systolic and diastolic blood pressure, pulse rate, total cholesterol, HDL cholesterol, glycosylated hemoglobin, and C-reactive protein. Logistic regression models, controlling for age and sex, are used to predict high-risk for each biomarker among Mexico-born Mexicans, Mexico-born Mexican-Americans, and U.S.-born Mexican-Americans. Results show that Mexico-born Mexicans exhibit higher biological risk for systolic blood pressure, pulse rate, low HDL cholesterol, glycosylated hemoglobin, and inflammation than Mexico-born and U.S.-born Mexican-Americans. Additionally accounting for socioeconomic status and health behaviors did not explain differences in high-risk among Mexican-born Mexicans.

